# Modeling of table grape soluble solids content, titratable acidity and pH prediction during storage based on Vis-NIR spectroscopy

**DOI:** 10.3389/fpls.2025.1723949

**Published:** 2025-12-01

**Authors:** Ke He, Yuan Su, Lei He, Can Hu, Jianfei Xing, Naimov Alisher

**Affiliations:** 1 College of Mechanical and Electronic Engineering, Northwest A & F University, Yangling, China; 2 College of Enology, Northwest A & F University, Yangling, China; 3 Mechanical Equipment Research Institut, Xinjiang Academy of Agricultural Reclamation Science, Shihezi, China; 4 College of Mechanical and Electrical Engineering, Tarim University, Alar, China; 5 College of engineering, China Agricultural University, Beijing, China; 6 Institute of Botany, Plant Physiology and Genetics, Academy of Sciences, Dushanbe, Tajikistan

**Keywords:** Vis-NIR, table grape, ssc, Ta, pH, prediction model

## Abstract

**Introduction:**

The soluble solids content (SSC), titratable acidity (TA), and pH are key indicators for evaluating the quality of table grapes during storage. Conventional detection methods are typically destructive and time-consuming. To address this limitation, visible-near infrared (Vis-NIR) spectroscopy was employed in this study to enable rapid and non-destructive quality assessment of fresh table grapes throughout the storage period.

**Methods:**

*Seedless White* table grape samples were analyzed within the 200–1100 nm spectral range, and calibration models for key quality parameters (SSC, TA, and pH) were established. Three machine learning algorithms, partial least squares regression (PLSR), support vector machine (SVM), and extreme learning machine (ELM), were employed to develop spectral prediction models based on characteristic wavelengths selected using different feature extraction strategies, including the successive projection algorithm (SPA), uninformative variable elimination (UVE), and competitive adaptive reweighted sampling (CARS).

**Results:**

The results demonstrate that the SNV-CARS-SVM models achieved excellent performance in predicting SSC with a root mean square errors (RMSEP) of 0.673, a coefficient of determination for the prediction data set (*Rp*) of 0.928 and an RPD of 3.311. Similarly, the SNV-SPA-SVM models exhibited excellent predictive accuracy for TA, yielding an RMSEP of 0.553, an Rp of 0.873, and an RPD of 2.662. Good performances were achieved with *Rp* of 0.758 and RMSEP of 0.113 with the SNV-CARS-PLSR model for pH.

**Discussion:**

This study, for the first time, utilized Vis-NIR spectroscopy to achieve the simultaneous and rapid determination of multiple quality attributes in table grapes, providing a novel and efficient strategy for real-time and non-destructive quality evaluation during storage. The proposed approach showed considerable potential for rapid quality assessment and postharvest management of grapes. Future research will focus on expanding the diversity of grape cultivars and investigating various storage conditions to improve the robustness and transferability of the predictive model, thereby promoting the industrial validation and practical application of Vis-NIR spectroscopy in fruit quality monitoring.

## 1 Introduction

Grape is an important economic crop with dual purposes serving both fresh consumption and processing. It is rich in glucose, fructose, organic acids, amino acids, and essential minerals, which together contribute to its high nutritional value. The flavonoids and anthocyanins in grape skins possess strong antioxidant and anti-aging properties (Zeng et al., 2024; Ma et al., 2024). According to data from the China Agricultural Yearbook, the China vineyard area reached approximately 0.7 million hectares in 2020, encompassing grapes cultivated for fresh consumption, winemaking, drying, and juice production (Li et al., 2021). Meanwhile, China’s table grape export volume has reeched around 375,000 tons in 2022, ranking third globally, which demonstrating the vigorous development of the grape industry (Liu et al., 2024).

Table grape quality is a crucial factor influencing consumer preferences and market value. High-quality table grapes not only exhibit superior sensory attributes but also possess enhanced nutritional characteristics. In general, grape quality can be classified into external and internal physicochemical attributes. The internal quality parameters mainly include soluble solids content (SSC), titratable acidity (TA), and pH (Wu et al., 2024). Soluble solids content (SSC) reflects the sweetness and nutritional status of the fruit, titratable acidity (TA) affects flavor balance, and pH is closely related to storage stability and sensory perception. At present, the internal quality of grapes is primarily evaluated through conventional chemical analysis, including acid-base titration, refractometry, and Fehling solution titration. Although these methods provide high accuracy, they are destructive, labor-intensive, time-consuming, and prone to human error, thereby reducing overall efficiency (Guo et al., 2015; Ma et al., 2018; Pissard et al., 2021). Therefore, developing a rapid, accurate, and non-destructive method for determining SSC, TA, and pH in table grapes is crucial for quality assessment and postharvest management.

With the rapid development of agricultural information technologies, spectral analysis has become a powerful tool for non-destructive fruit quality assessment due to its speed, convenience, and reliability. Visible and near-infrared (Vis-NIR) spectroscopy enables quantitative prediction of internal quality parameters by analyzing the optical properties of fruit tissues (Roberto et al., 2018; Ncama et al., 2018). For example, in studies predicting grape quality, Dambergs et al. (2006) applied Vis-NIR spectroscopy to determine anthocyanins, pH, and SSC in wine grapes across multiple cultivars, vintages, and regions, developing predictive models with robust performance. Similarly, Xiao et al. (2018) employed Vis-NIR spectra to classify five ripening stages of different grape varieties, achieving classification accuracies of 90% and 100% for “Manicure Finger” and “Ugni Blanc,” respectively. These findings highlight the feasibility of near-infrared spectroscopy for grape quality assessment. The table grapes undergo a storage period from harvest to sale, during which their internal quality may change. However, most existing studies have focused on predicting the multiple qualities of table grapes at maturity (Xiao et al., 2018), while few studies on the detection of multiple parameters in table grapes during storage remain limited.

**Figure 1:**
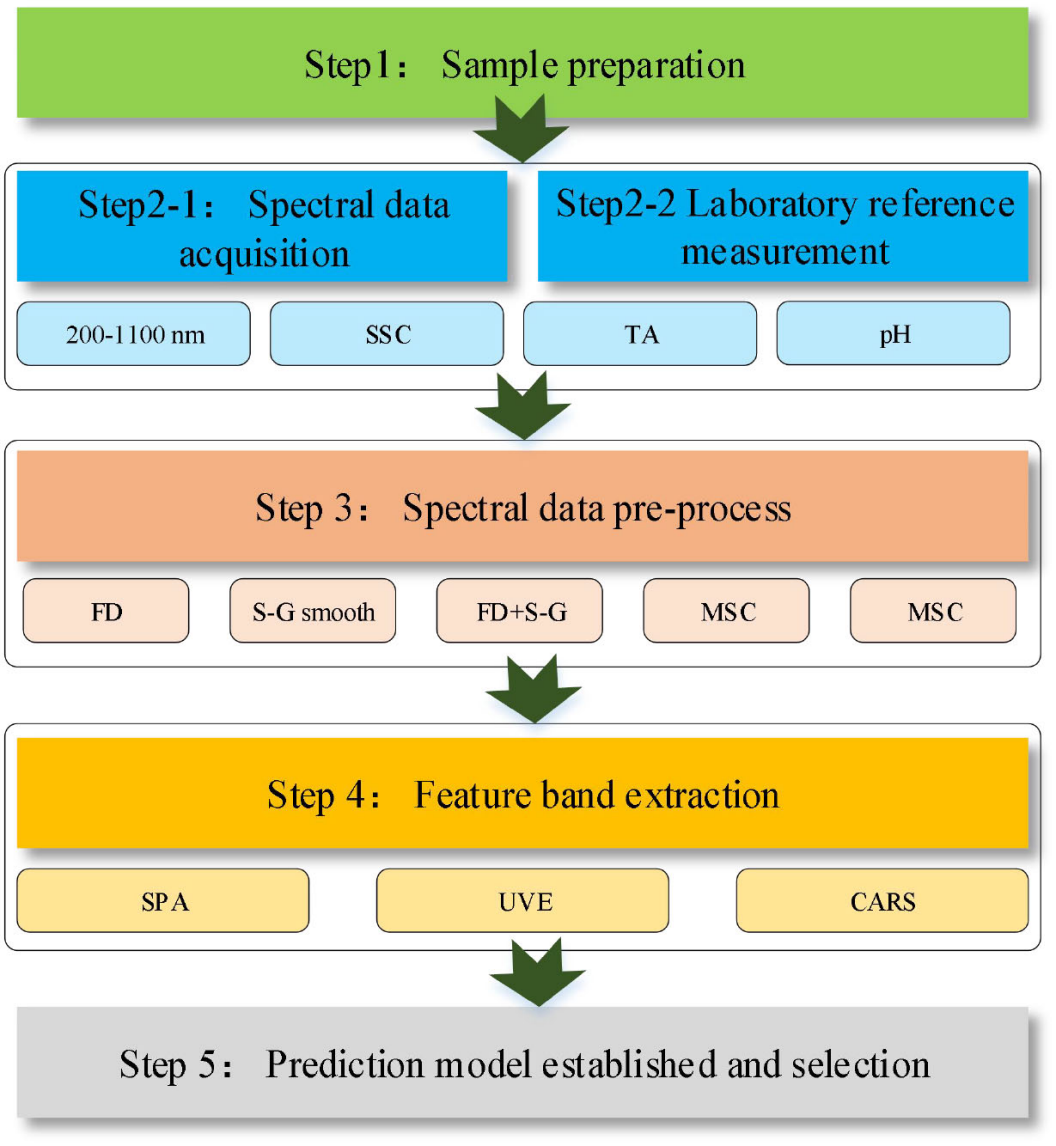
Schematic diagram illustrating the workflow of data processing.

In light of the aforementioned limitations, this study aims to develop a rapid and non-destructive method for the simultaneous determination of multiple quality parameters in table grapes during storage using Vis-NIR spectroscopy. The specific objectives of this research are: (1) to acquire Vis-NIR spectral data alongside corresponding reference measurements of table grape quality parameters during storage; (2) to identify the most effective data preprocessing method for enhancing model performance through comparative analysis; and (3) to optimize predictive models by evaluating different modeling algorithms and wavelength selection strategies. This study seeks to advance table grape quality assessment from conventional destructive analyses toward intelligent, non-destructive evaluation, thereby improving quality control efficiency during table grape storage. The main steps of evaluation of quality parameters in table grape samples by Vis-NIR were schematically shown in **Figure 1**.

## 2 Materials and methods

### 2.1 Experiment materials

The experimental material used in this study consisted of *Seedless White* table grapes (Vitis vinifera L.) harvested from the Caoxinzhuang Experimental Farm (34°18′0″ N; 108°5′23.9″ E) of Northwest A&F University in Yangling, Shaanxi Province. To prevent damage to the berries, individual grapes were carefully excised from the clusters using pruning shears, leaving a short section of the pedicel attached to minimize moisture loss. Uniform, undamaged berries of similar size were selected and divided into three groups. Grapes were stored for 15 days under three temperature conditions: cold storage (2.7 °C), refrigeration (10.0 °C), and room temperature (20.6 °C). For each measurement, nine berries per group were used for spectral data acquisition, while twenty berries were used to determine soluble solids content (SSC), titratable acidity (TA), and pH. In total, 145 sets of SSC, TA, pH, and spectral data were collected.

### 2.2 Vis-NIR spectroscopy detection acquisition

**Figure 2:**
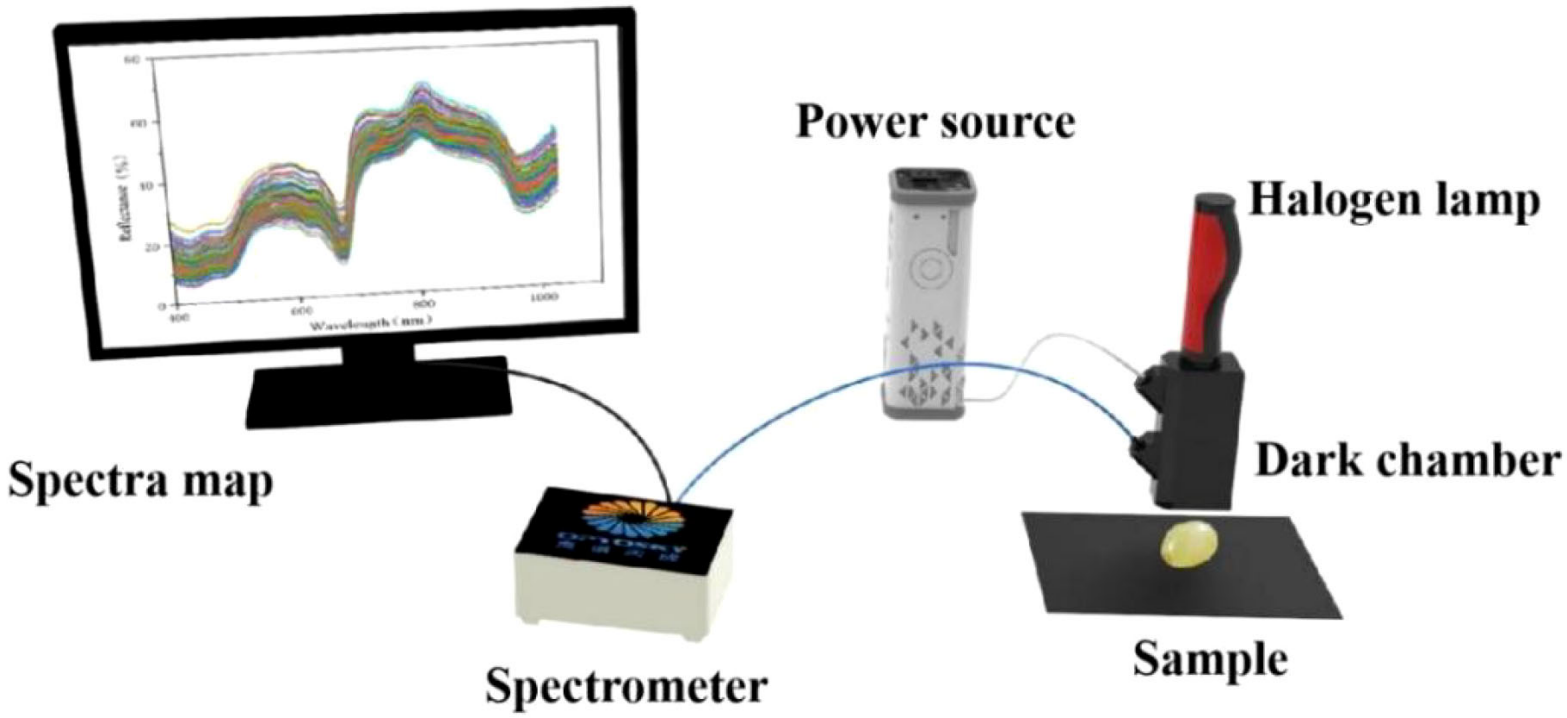
The Vis-NIR spectroscopy data acquisition system.

The spectral data were collected using a ATP3030 spectrometer system (Optosky Photonics Inc., Xiamen, China). The spectrometer system includes a halogen lamp (HL-2000), a dark chamber, a computer, and a spectrometer in the 200–1100 nm wavelength range with a resolution of 0.5 nm. The diffuse reflection whiteboard (WR-D97) and an optical fiber are also necessary for the experiment. The overall composition of the experimental system is shown in **Figure 2**. Data were acquired using Optosky Spectra software (V3.1.25) with a 1 ms integration time. Black and white reference measurements were performed and calibrated prior to data acquisition. Due to significant noise at the spectral front, only data within the 400–1100 nm range were retained, yielding 1508 variables per spectrum curve. Grape spectral data were collected under illumination from a tungsten halogen lamp. For each grape sample, spectra were acquired three times, and the mean value was used as the final spectral data for analysis.

### 2.3 Laboratory reference measurement

The grape samples from each group were separated into skin, flesh, and seeds. The flesh was pressed and filtered through four layers of gauze to obtain grape juice, which was subsequently used to determine the soluble solids content (SSC), pH, and titratable acidity (TA). The SSC was measured using a digital handheld refractometer (ATAGO PAL-1, Japan). Each measurement was performed in triplicate, and the mean value was recorded for analysis (Su et al., 2025). The pH of the grape juice was determined using a calibrated pH meter. Prior to measurement, the instrument was standardized with buffer solutions of known pH values, and the electrode was subsequently immersed in the juice sample. The pH value was recorded after stabilization. Each sample was measured in triplicate, and the mean value was calculated for analysis (Liu et al., 2024). The titratable acidity (TA) of each sample was determined by potentiometric titration following the *OIV* “Wine and Grape Juice Analysis Method,” using a Mettler automatic potentiometric titrator. Prior to analysis, the titrator was calibrated. A 2 mL aliquot of grape juice was transferred into a beaker, to which 50 mL of distilled water was added. The electrode and magnetic stirrer were immersed in the solution, and titration was carried out with a standard sodium hydroxide solution. TA was expressed as tartaric acid equivalents. Each sample was measured in triplicate, with a maximum measurement error of 0.5 mL, and the mean value was recorded. Blank determinations were performed simultaneously, and the final TA values were corrected accordingly (Xing et al., 2025; Zhou et al., 2024).

### 2.4 Spectral data pre-process method

The collected spectral data are affected by multiple factors, including environmental conditions, instrument performance, and random noise, which may introduce redundant or irrelevant information and consequently reduce model stability and accuracy. To mitigate these effects and improve the reliability of subsequent analyses, preprocessing of the raw spectral data is therefore essential. In this study, five preprocessing methods were applied, including first derivative (FD), Savitzky-Golay convolutional smoothing (S-G), standard normal variate (SNV), and multivariate scatter correction (MSC). First derivative processing calculates the first derivative of the spectral data to enhance spectral features and minimize baseline shifts and offsets. (Ferreira et al., 2018). This method is particularly effective for complex spectral datasets, as it improves data accuracy and reliability, although it may also amplify noise. The S-G smoothing method, which is based on the least-squares polynomial fitting principle, is widely used for spectral smoothing (De-Lima et al., 2018). By fitting polynomials to local segments of the data, this method smooths the spectral curve and suppresses random noise, resulting in a more continuous and stable signal. The SNV transformation corrects for systematic shifts and scaling variations in spectral data, thereby standardizing the spectra and improving their comparability (Caramês et al., 2019). The MSC method effectively eliminates spectral distortions caused by variations in scattering intensity, thereby enhancing the correlation between spectral data and target attributes (Guo et al., 2011).

### 2.5 Feature band extraction method

The Vis-NIR spectroscopy produces a large volume of spectral data; however, such high-dimensional datasets are not ideal for real-time data acquisition and processing. Therefore, selecting a subset of optimal wavelengths that effectively represent the full spectrum is essential for reducing data dimensionality and improving the computational efficiency of modeling. In this study, three wavelength selection methods were employed. The successive projection algorithm (SPA) is an effective multivariate calibration method for variable selection, capable of identifying characteristic spectral bands and reducing collinearity among variables (Lu et al., 2019). The uninformative variable elimination (UVE) is a feature wavelength selection method that uses partial least squares regression coefficients to assess the importance of each wavelength. The UVE method accounts for both noise and physicochemical information during feature wavelength selection, thereby minimizing the risk of choosing variables that are insensitive to the target components (Zhang et al., 2020). The competitive adaptive reweighted sampling (CARS) efficiently reduces the dimensionality of the spectral data and identifies key variables to enhance model predictive performance (Zhang et al., 2022).

### 2.6 Model calibration

In this study, three regression algorithms, partial least squares regression (PLSR), support vector machine (SVM), and extreme learning machine (ELM), were applied and compared to develop predictive models for the quality parameters of *Seedless White* grape samples. The PLSR algorithm transforms raw spectral data into a set of orthogonal and independent latent variables that preserve the essential information of the original spectra. This approach effectively addresses collinearity in spectral data, suppresses noise interference, and allows simultaneous modeling of multiple response variables (Su et al., 2019). In the PLSR model, cross-validation was employed to determine the optimal number of latent variables. The SVM algorithm, a nonlinear modeling technique grounded in statistical learning theory, constructs an optimal hyperplane in a high-dimensional feature space to perform regression or classification. Its capability to handle nonlinear, high-dimensional, and highly correlated data offers strong generalization performance for complex spectral datasets (Yu et al., 2023).In this study, the support vector machine (SVM) employed the radial basis function (RBF) as its kernel, and the hyperparameters c and g were optimized using a grid search method. The ELM algorithm, a single-hidden-layer feedforward neural network model, randomly generates input-to-hidden layer weights and analytically determines output-layer weights, enabling rapid learning and prediction. This approach achieves high predictive accuracy while substantially improving computational efficiency, making it particularly suitable for large-scale spectral data analysis (Sun et al., 2023). By comparing these three algorithms, the most suitable model for predicting grape quality parameters was determined.

### 2.7 Evaluation of the indicators

To comprehensively assess a model’s predictive performance, commonly used metrics include the correlation coefficient of the calibration set (*R*_C_), the correlation coefficient of the prediction set (*R*_P_), the root mean square error of calibration (RMSEC), the root mean square error of prediction (RMSEP), and the ratio (RPD) of the prediction set standard deviation to RMSEP (Kamruzzaman et al., 2016; Xu et al., 2016; Zheng et al., 2019). For a spectral prediction model to achieve high predictive accuracy, the evaluation criteria typically include: (1) maximizing the correlation coefficients of the calibration and prediction sets (*R*_C_ and *R_P_*) while minimizing the corresponding root mean square errors (RMSEC and RMSEP); (2) ensuring that the correlation coefficients and root mean square errors of the calibration and prediction sets are comparable, with *R*_C_ slightly exceeding *R*_P_ and RMSEC slightly less than RMSEP; and (3) attaining the highest ratio of performance to deviation (RPD) (Lu et al., 2019; Khoshnoudi-Nia and Moosavi-Nasab, 2019).

## 3 Results and discussion

### 3.1 Statistics of measured reference values

Developing spectral prediction models with high accuracy and reliability requires that the calibration and prediction datasets satisfy specific distributional criteria for reference measurements. The range of reference values in the calibration set should fully cover that of the prediction set. Furthermore, the mean and standard deviation of reference values in the calibration set should be slightly higher than or comparable to those in the prediction set (Su et al., 2025). In this study, 145 samples were divided into calibration and prediction sets at a ratio of 3:1 using the gradient concentration method to satisfy these requirements. The results were shown in **Table 1**, where the calibration set comprised 109 samples and the prediction set included 36 samples. The reference measurement ranges for SSC in the calibration and prediction sets were 9.23-22.73% and 11.40-22.07%, respectively; for TA in the calibration and prediction sets were 3.87-9.79 g/L and 3.96-9.79 g/L; and for pH in the calibration and prediction sets were 3.22-4.10 and 3.06-4.59, respectively. These results indicated that the calibration set fully encompassed the prediction set.

**Table 1:** Reference measurement of seedless white grape.

Parameter	Subsets	Sample number	Range	Mean	Standard deviation
SSC(%)	Calibration set	109	9.23-22.73	17.34	2.34
	Prediction set	36	11.4-22.07	17.35	2.23
	Total samples	145	9.23-22.73	17.35	2.30
TA(g/L)	Calibration set	109	3.87-9.79	5.91	1.44
	Prediction set	36	3.96-9.72	5.93	1.47
	Total samples	145	3.87-9.79	5.92	1.46
pH	Calibration set	109	3.06-4.59	3.82	0.20
	Prediction set	36	3.22-4.10	3.82	0.19
	Total samples	145	3.06-4.59	3.82	0.20

### 3.2 Spectral characterization of table grape

**Figure 3:**
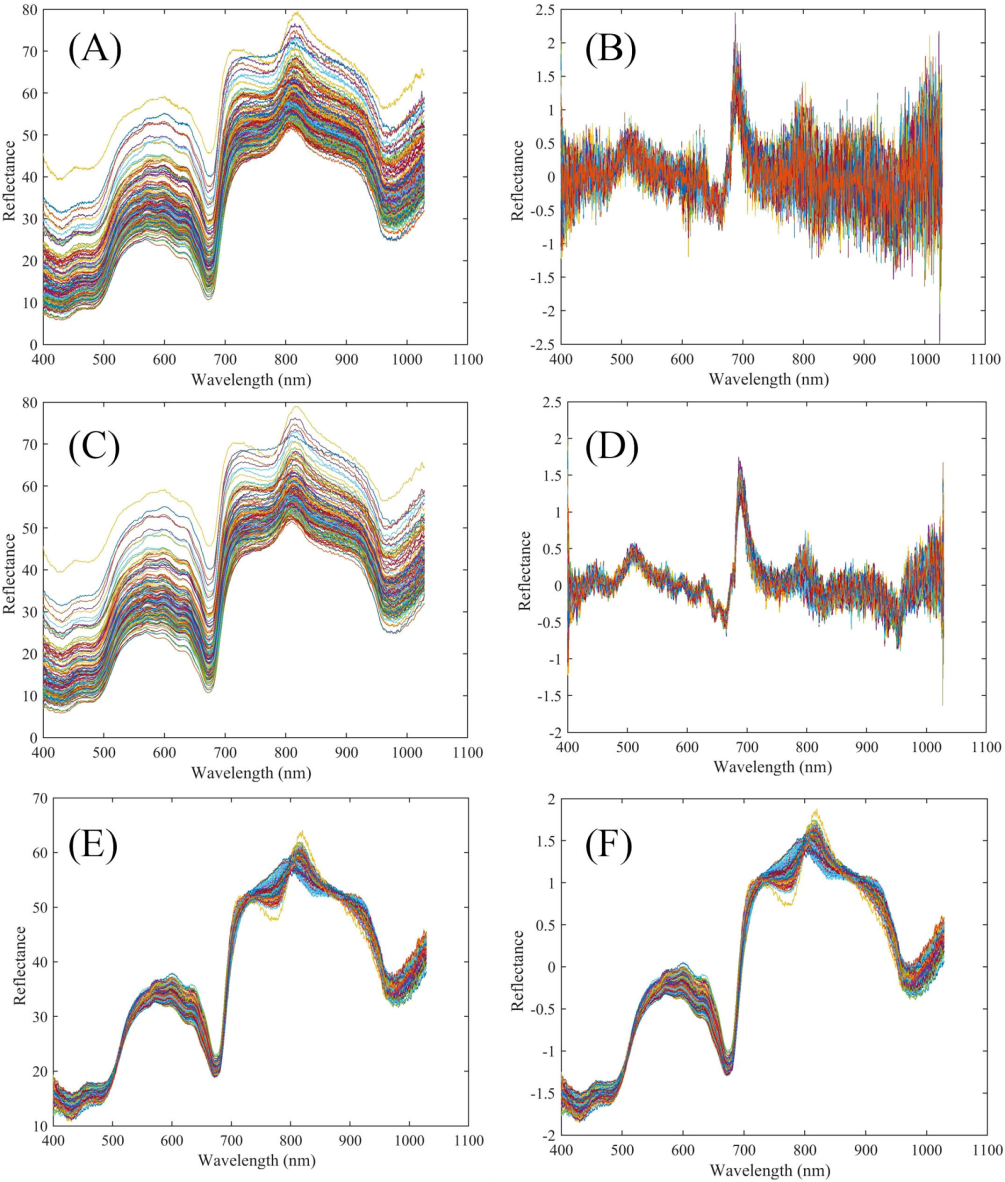
Grapes spectral reflectance curves **(A)** Raw spectral curve of grapes **(B)** FD preprocess spectral curve of grapes **(C)** S-G preprocess spectral curve of grapes **(D)** FD+S-G preprocess spectral curve of grapes **(E)** MSC preprocess spectral curve of grapes **(F)** SNV preprocess spectral curve of grapes.

**Figure 3** presents the original and preprocessed spectral curves of seedless white grapes. The spectral profiles of samples stored for different durations exhibit similar patterns without any obvious abnormal peaks, indicating that the spectral data of all 145 grape samples are consistent and free from significant anomalies. Nevertheless, variations in spectral reflectance intensity were observed, indicating the need for multivariate data analysis to clarify the relationship between near-infrared spectra and texture characteristics.Three prominent peaks were observed at 530–630 nm, 720 nm, and 810 nm, accompanied by two valleys at 670 nm and 970 nm. The yellow-green skin color of *Seedless White* grapes corresponds to the peak between 580 and 630 nm. The valley at 670 nm lies within the chlorophyll absorption region and may be associated with the absorption of carotenoids and chlorophyll. The absorption at 810 nm may be attributed to temperature-related optical path corrections, whereas the absorption at 970 nm corresponds to the stretching vibrations of carbohydrates and O-H bonds in water (Lin et al., 2024).

### 3.3 Prediction models using full spectral range

In this study, five spectral preprocessing algorithms were applied to establish quantitative relationships between the reflectance spectral data matrix (X) and the reference variable (Y) across the entire spectral range via partial least squares regression (Xu et al., 2016). A linear regression model incorporating chemical composition and full-spectrum information was developed to predict the SSC, TA, and pH of seedless white grapes. As shown in **Table 2**, the FD preprocess method decreased the *Rp* and RPD values of the prediction model due to amplified spectral noise. In contrast, S-G smoothing, MSC, and SNV preprocessing methods yielded models with superior predictive performance compared with the model based on the raw spectra. Among these models, the model developed using SNV preprocessing achieved the highest prediction accuracy. The correlation coefficients (*Rc* and *Rp*), root mean square errors (RMSEC and RMSEP), and ratio of performance to deviation (RPD) for the SSC prediction model were 0.949, 0.897, 0.736, 0.984, and 2.266, respectively; for the TA prediction model, they were 0.877, 0.868, 0.690, 0.739, and 1.997, respectively; and for the pH prediction model, they were 0.759, 0.756, 0.129, 0.124, and 1.513, respectively. These findings indicated that the PLSR model developed using the SNV-preprocessed matrix provided the best predictive performance for determining the quality parameters of *Seedless White* grapes. Therefore, the SNV preprocessing methods could be effectively applied in subsequent analyses to extract valuable information regarding grape quality attributes.

**Table 2:** Results of PLSR models based on different spectral pretreatments.

Parameter	Pre-processing	Calibration set		Prediction set		
		*R*_C_	RMSEC	*R*_P_	RMSEP	RPD
SSC	RAW	0.880	1.111	0.839	1.240	1.799
	FD	0.884	1.098	0.772	1.514	1.473
	S-G	0.939	0.806	0.859	1.149	1.940
	FD+S-G	0.886	1.096	0.744	1.576	1.416
	MSC	0.922	0.903	0.877	1.094	2.038
	**SNV**	**0.949**	**0.736**	**0.897**	**0.984**	**2.266**
TA	RAW	0.870	0.709	0.835	0.810	1.820
	FD	0.977	0.307	0.828	0.831	1.775
	S-G	0.885	0.668	0.845	0.818	1.804
	FD+S-G	0.970	0.347	0.828	0.854	1.727
	MSC	0.864	0.722	0.836	0.806	1.830
	**SNV**	**0.877**	**0.690**	**0.868**	**0.739**	**1.997**
pH	RAW	0.857	0.102	0.716	0.141	1.333
	FD	0.807	0.121	0.469	0.201	0.931
	S-G	0.722	0.138	0.693	0.140	1.337
	FD+S-G	0.927	0.074	0.678	0.150	1.249
	MSC	0.743	0.133	0.760	0.121	1.545
	**SNV**	**0.759**	**0.129**	**0.756**	**0.124**	**1.513**

Bold represents the optimal model.

### 3.4 Selection of effective wavelengths

**Figure 4:**
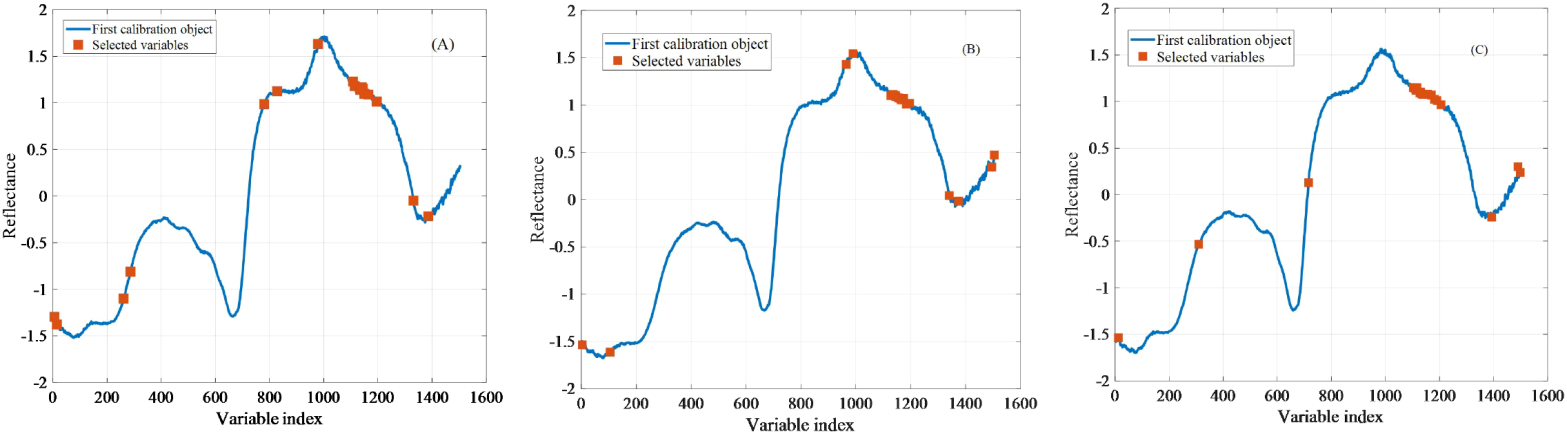
The effective variables of grape quality parameters selected by SPA method. The optimal subset of SSC, TA and pH contains 20, 20 and 20 variables respectively, displayed as solid rectangles in **(A–C)**.

When using the SPA method to screen feature wavelengths, the maximum number of selected variables was set to 30. **Figure 4** illustrated the feature wavelengths selected by the SPA for SSC, TA and pH in grapes. The corresponding wavelengths associated with SSC were 615, 261, 287, 780, 828, 979, 1108, 1114, 1133, 1135, 1138, 1140, 1142, 1144, 1150, 1165, 1196, 1331, and 1386 nm. The wavelengths associated with TA were 3, 105, 965, 990, 1127, 1136, 1138, 1144, 1146, 1156, 1163, 1165, 1170, 1174, 1184, 1196, 1340, 1374, 1495, and 1505 nm. The wavelengths associated with pH were 12, 309, 716, 1103, 1109, 1113, 1117, 1127, 1131, 1135, 1139, 1149, 1156, 1170, 1181, 1191, 1206, 1393, 1490, and 1499 nm. Notably, multiple variables within the 1000–1200 nm wavelength range were consistently selected for all three quality parameters, suggesting that this spectral region plays a critical role in constructing predictive models for grape quality assessment (Yang et al., 2022).

**Figure 5:**
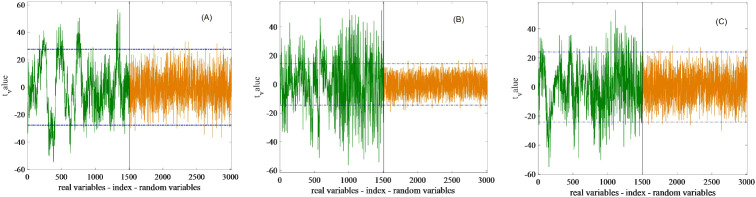
The effective variables of grape quality parameters selected by UVE method. The optimal subset of SSC, TA and pH contains 275, 98 and 239 variables respectively, displayed in **(A–C)**.

**Figure 5** shows the distribution of feature wavelengths selected by the UVE method after SNV preprocessing. The horizontal axis corresponds to the spectral and noise matrix, whereas the vertical axis represents the stability *t*-value. Two parallel lines indicate the threshold limits: variables with stability values between these lines were excluded, whereas those exceeding the threshold were retained as feature wavelengths. After removing irrelevant variables, the number of feature variables associated with SSC, TA, and pH was reduced to 275, 98, and 239, respectively. Notably, the UVE-selected feature variables include wavelengths in the 1000–1200 nm range and meanwhile span nearly the entire spectral range.

**Figure 6:**
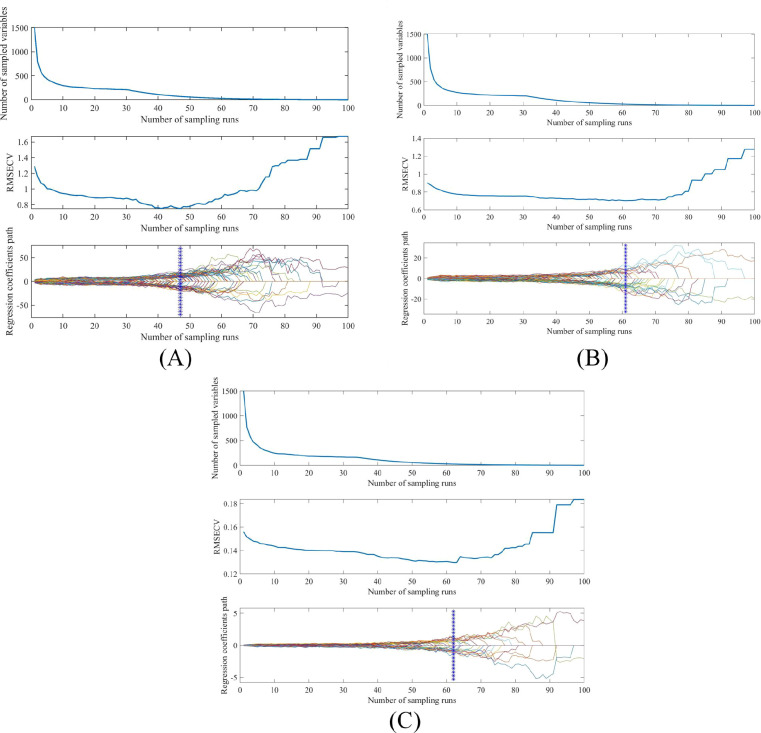
The effective variables of grape quality parameters selected by CARS method. The optimal subset of SSC, TA and pH contains 57, 27 and 25 variables respectively, displayed as solid rectangles in **(A–C)**.

**Figure 6** illustrates the selection of characteristic wavelengths for grape quality parameters via the CARS algorithm. It depicts the relationship among the number of sampling runs, the number of selected wavelength variables, RMSECV values, and regression coefficient trajectories (Cheng et al., 2020; Fan et al., 2021). As the number of sampling runs increases, the efficiency of feature variable selection improves substantially, progressing from coarse to fine screening. The RMSECV value attains its minimum at the 48th sampling run. During the 1st to 48th sampling runs, the RMSECV value decreases, followed by an upward trend from the 49th to the 100th run, indicating that the CARS algorithm may have eliminated some critical information related to SSC. Ultimately, the CARS algorithm selected 57, 27, and 25 wavelengths for SSC, TA, and pH, respectively.

### 3.5 Prediction models using effective wavelengths

This study compares the performance of SVM, PLSR, and ELM models to determine the most effective modeling approach. Based on the prediction of quality parameters for seedless white grapes (**Table 2**), PLSR and SVM models constructed with feature wavelengths exhibited superior performance compared to the ELM model. ELM is fundamentally a linear least squares solution, rendering it susceptible to overfitting or oscillation when the input data contain substantial noise or multicollinearity. Furthermore, extreme learning machines exhibit limited generalization ability and stability due to the random initialization of input weights and hidden layer biases, as well as their sensitivity to noise and redundant features (Tang et al., 2016; Huang et al., 2012). In contrast, PLSR and SVM incorporate more sophisticated parameter optimization and feature selection mechanisms, resulting in greater modeling accuracy and robustness. In addition, Wan et al. (2020) reported that the predictive performance of nonlinear regression models generally exceeds that of linear models, with SVM models often outperforming PLSR models. Therefore, for the prediction of SSC and TA, the SVM model achieved the highest predictive performance.

**Figure 7:**
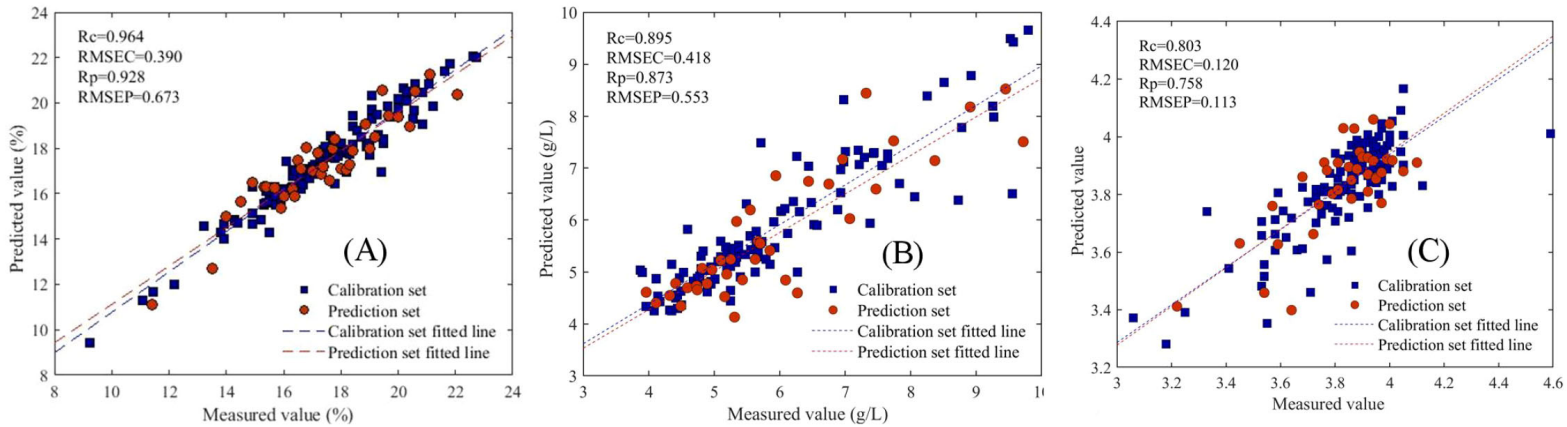
Correlation between measurement values and predicted values in the calibration set and prediction set **(A)** SSC **(B)** TA **(C)** pH.

The selection of feature wavelengths by different algorithms markedly influences model predictive performance (**Table 3**). Specifically, the SNV-CARS-SVM model achieved the most accurate prediction of SSC, with correlation coefficients (*Rc* and *Rp*) of 0.964 and 0.928 for the calibration and prediction sets, and root mean square errors (RMSEC and RMSEP) of 0.390 and 0.673, respectively. The model’s RPD value reached 3.311. For TA prediction, the SNV-SPA-SVM model performed optimally, with *Rc* and *Rp* values of 0.895 and 0.893, RMSEC and RMSEP of 0.418 and 0.553, and an RPD value of 2.622. The SNV-CARS-PLSR model exhibited the highest predictive performance for pH, with *Rc* and *Rp* values of 0.803 and 0.758, RMSEC and RMSEP of 0.120 and 0.113, and an RPD value of 1.655. The optimum model of SSC, TA and pH was displayed more intuitively by the scatter plots of **Figure 7**.

**Table 3:** Prediction of SSC, TA and pH based on different method in characteristic wavelengths.

Parameter	Wavelength selection	Number of wavelength	Model	Calibration set		Prediction set		
				*R*_C_	RMSEC	*R*_P_	RMSEP	RPD
SSC	SPA	20	PLSR	0.831	1.297	0.785	1.384	1.611
			SVM	0.865	1.373	0.812	1.680	1.327
			ELM	0.957	0.675	0.805	1.828	1.219
	UVE	275	PLSR	0.880	1.107	0.838	1.228	1.816
			SVM	0.900	1.047	0.840	1.427	1.562
			ELM	0.958	0.668	0.787	2.379	0.937
	CARS	57	PLSR	0.956	0.680	0.914	0.900	2.478
			**SVM**	**0.964**	**0.390**	**0.928**	**0.673**	**3.311**
			ELM	0.969	0.572	0.870	1.331	1.675
TA	SPA	20	PLSR	0.832	0.798	0.873	0.716	2.060
			**SVM**	**0.895**	**0.418**	**0.873**	**0.553**	**2.662**
			ELM	0.950	0.445	0.810	0.978	1.505
	UVE	98	PLSR	0.872	0.704	0.867	0.745	1.979
			SVM	0.982	0.083	0.710	1.122	1.314
			ELM	0.934	0.511	0.808	1.132	1.300
	CARS	27	PLSR	0.890	0.655	0.904	0.637	2.314
			SVM	0.914	0.340	0.856	0.623	2.366
			ELM	0.974	0.321	0.853	0.981	1.500
pH	SPA	20	PLSR	0.743	0.135	0.755	0.114	1.640
			SVM	0.707	0.210	0.677	0.162	1.154
			ELM	0.902	0.087	0.619	0.291	0.643
	UVE	239	PLSR	0.758	0.132	0.746	0.115	1.626
			SVM	0.812	0.144	0.561	0.147	1.272
			ELM	0.930	0.073	0.656	0.187	1.000
	CARS	25	**PLSR**	**0.803**	**0.120**	**0.758**	**0.113**	**1.655**
			SVM	0.759	0.180	0.691	0.170	1.100
			ELM	0.946	0.065	0.603	0.270	0.693

Bold represents the optimal model.

Among the three quality parameters, pH prediction was less accurate than those of SSC and TA, primarily owing to differences in chemical properties and spectral responses. SSC and TA are closely associated with soluble sugars, organic acids, and other constituents exhibiting distinct absorption characteristics in the near-infrared region (e.g., vibrations and stretching of O-H, C-H, and C=O groups), rendering their concentration changes readily detectable in the spectra (Li et al., 2006). In contrast, pH reflects hydrogen ion activity rather than the concentration of specific chemical groups, yielding a more indirect and nonlinear relationship with spectral signals (Nicolaï et al., 2007). Furthermore, pH is influenced by multiple factors, including the type of organic acids, buffer systems, and ionic strength, which often exhibit weak or overlapping near-infrared absorption features (Koashi et al., 2003). Consequently, the spectral response of pH is weaker and less distinct, limiting the predictive capability of models compared with SSC and TA.

The table grape variety used in this study was *Seedless White*, a typical white cultivar with a thin peel, tender flesh, and sweet flavor. Accordingly, the developed prediction model primarily reflects quality variations in white grapes under different storage conditions. Its applicability to other cultivars, particularly red or purple types (e.g., *Kyoho* and *Beauty Finger*), requires further validation due to their higher anthocyanin and phenolic contents and distinct metabolic behaviors during storage. Future research will expand the sample set to include multiple varieties, color types, and production regions, enabling a more robust and generalizable predictive model.

While this study considered conditions representative of typical postharvest handling, grapes in practice may experience more complex environments, such as controlled-humidity or controlled-atmosphere storage, or fluctuating temperature and humidity. These factors can significantly affect physicochemical properties, sensory attributes, and metabolic activity. Further investigation into the influence of environmental factors, including temperature, humidity, gas composition, and light exposure, will support the development of a more comprehensive and accurate model, providing a scientific basis for optimizing storage management and preserving grape quality.

## 4 Conclusion

The soluble solids content (SSC), titratable acidity (TA), and pH are critical indicators for assessing the quality of table grapes during storage. This study aimed to develop a model for the rapid and non-destructive detection of these parameters in table grapes during storage using visible near infrared (Vis-NIR) spectroscopy. Through comparative experiments involving different spectral preprocessing techniques and feature wavelength selection algorithms, the optimized model achieved fast and non-destructive prediction of SSC, TA, and pH in *Seedless White* grapes, demonstrating superior performance. This research provides a new approach for rapid, non-destructive, and high-precision quality assessment of table grapes during storage. The findings offer practical significance for promoting the sustainable development of the grape industry.

## Data Availability

The raw data supporting the conclusions of this article will be made available by the authors, without undue reservation.
